# The Role of Selenium in Arsenic and Cadmium Toxicity: an Updated Review of Scientific Literature

**DOI:** 10.1007/s12011-019-01691-w

**Published:** 2019-03-15

**Authors:** Iwona Zwolak

**Affiliations:** Laboratory of Oxidative Stress, Centre for Interdisciplinary Research, The John Paul II Catholic University of Lublin, Konstantynów 1 J, 20-708 Lublin, Poland

**Keywords:** Arsenic, Cadmium, Selenium interactions, Antioxidants, Nrf2 factor, Toxicity

## Abstract

Arsenic (As) and cadmium (Cd) are elements arousing major public health concerns associated with environmental pollution, high toxicity potential, and carcinogenic nature. However, selenium (Se) at low doses and incorporated into enzymes and proteins has antioxidant properties and protects animals and humans from the risk of various diseases. It also has an exceptionally narrow range between necessary and toxic concentrations, which is a well-known hindrance in its use as a dietary supplement. The present article aims to update and expand the role of Se in As and Cd toxicity discussed in our earlier paper. In general, recent reports show that Se, regardless of its form (as selenite, selenomethionine, nanoSe, or Se from lentils), can reduce As- or Cd-mediated toxicity in the liver, kidney, spleen, brain, or heart in animal models and in cell culture studies. As was suggested in our earlier review, Se antagonizes the toxicity of As and Cd mainly through sequestration of these elements into biologically inert complexes and/or through the action of Se-dependent antioxidant enzymes. An increase in the As methylation efficiency is proposed as a possible mechanism by which Se can reduce As toxicity. However, new studies indicate that Se may also diminish As or Cd toxicity by activation of the Nrf2 pathway. In addition, this paper discusses possible signs of Se toxic effects, which may be a challenge for its future use in the therapy of As and Cd poisoning and provide future directions to address this issue.

## Introduction

Arsenic (As) and cadmium (Cd) are toxic elements and well-known human carcinogens. Due to natural factors and human activities, they both contribute to environmental pollution, posing a health risk for animals and humans mainly through contaminated water and food. Because they are odorless and tasteless, their excessive presence in the environment is often unrecognizable and causes endemic human poisoning, whose most severe forms are known as itai-itai disease (chronic Cd poisoning) and chronic arsenicosis (arsenic poisoning), which is even more widespread. Adequate strategies have been implemented to reduce the environmental As and Cd exposure; however, due to, e.g., the persistent nature of these elements, their negative impact on human health is still being observed and documented by epidemiological studies, as described further in the next chapters. The conventional therapy of As and Cd poisoning involves the use of chelating agents, which unfortunately can be toxic themselves and induce side effects [1, 2]. Therefore, other supportive treatments such as high-protein diet or the use of nutrients like vitamins, probiotics, polyphenols, or essential elements are also proposed to help in reducing the deleterious effects of As and Cd [1–3].

Over the last decades, a number of environmental and experimental studies have been published, which looked into the potential use of essential mineral selenium (Se) in the treatment of As^III^/As^V^ and Cd toxicity, reflecting the enormous interest in this subject [4–9]. These works were reviewed in our previous paper [10], which included a literature search up to 2011. We suggested that the main mechanism of Se protection against As and Cd in animals is based on sequestration of these toxic elements into biologically inert complexes. The well-known antioxidant selenoenzymes such as glutathione peroxidase (GPx) and thioredoxin reductase (TrxR) also mediate Se-dependent detoxification of As and Cd, but they probably play a secondary role [10].

However, many new reports have been published since 2012, which justify the update. To this end, the present contribution highlights papers from 2012 to 2018 presenting the most recent data on Se interactions with As (especially with As^III^) and Cd. New evidence has emerged with regard to Se effects on As^III^- and Cd-induced toxicity in various animal organs and tissues, including the liver, brain, testes, heart, and immune cells. In particular, new important data are reported on the role of Se in As^III^- and Cd-mediated activation of stress signaling pathways such as endoplasmic reticulum (ER) stress or nuclear factor erythroid 2-related factor (Nrf2) pathways. In addition, given the double-edged sword nature of Se, concerns regarding the potential signs of Se toxicity are also presented in a separate chapter.

## Selenium and Its Health Effects in Humans and Animals

Se was identified to be essential for higher animals in 1957 in the research conducted by Schwarz and Foltz [11], who showed that dietary Se prevented liver necrosis in vitamin E-deficient rats, and the study carried out by Patterson et al. [12], who demonstrated that Se protected vitamin E-deficient chicks from exudative diathesis. In 1958, a relationship was reported between very low soil Se concentrations and white muscle disease, i.e., myopathy in calves and lambs [13]. However, only in 1973, the first biochemical role of Se was established when it was discovered to be an essential component of erythrocyte GPx, which is a powerful antioxidant enzyme [14, 15]. Five years later, Se in the GPx enzyme was identified to be in the form of selenocysteine (SeCys) [16]. Simultaneously, Keshan disease was the first human disease to be associated with Se deficiency [17], and Se was suggested as an essential trace element for humans [18]. Since then, Se deficiency has been linked directly or indirectly with a large variety of animal and human disorders described further in this chapter.

Se found in the human body mainly comes from dietary sources. Food that contributes most to Se intake includes meat, cereals, and seafood [19]. Forms of Se that are found in food nutrients include organic Se compounds such as selenomethionine (SeMet, mainly cereals and Se yeast) and selenocysteine (SeCys, animal food) and inorganic Se forms, i.e., selenite (contains Se in the + 4 oxidation state) added to dietary supplements and selenate (contains Se in the + 6 oxidation state), which is present in dietary supplements and water [20]. Se as selenite is also a common feed additive in commercial animal diets with accepted maximal total content of 0.5 mg Se/kg of complete feed [21]. Human Se intakes vary across different countries. In general, Se intakes are high in North America (93–134 μg day^−1^ for adults) [22], optimal in Western Europe (for example in France 64–52 μg day^−1^ in adults) [23], and low in Eastern European countries (30–40 μg day^−1^ in Poland) [24, 25]. More to the point, the daily dietary intakes of Se recommended by international agencies to avoid Se deficiency or excess vary depending on the criteria established by these institutions to assess the physiological requirement of Se. Thus, the United States National Academy of Sciences recommends dietary intake of 55 μg Se day^−1^ for adults based on the amount of Se needed for maximization of GPx activity in plasma [26]. The World Health Organization established lower recommended Se intakes of 26 and 34 μg day^−1^ for adult females and males, respectively, on the basis that two thirds of maximal GPx activity instead of the maximal value is sufficient to provide health protection [27]. Another institution, the European Food Safety Authority (EFSA) in the European Union, set adequate intake of 70 μg Se day^−1^ for adults on the basis that this amount of Se is required for the selenoprotein P concentration to level off [28]. In addition, some researchers postulate to set different values for Se requirements for inorganic and organic Se forms, which would be 2 and 30 μg/day (acceptable upper daily intakes), respectively [27].

The fulfillment of Se functions in animals mainly depends on the expression of selenoproteins. This small (25 selenoproteins in humans) but pivotal group of proteins includes among others glutathione peroxidases (GPxs) and TrxRs participating in redox state regulation and antioxidant defense, idothyronine deiodinases (DIOs) required for thyroid hormone synthesis and metabolism, GPx4 playing specific roles in spermatogenesis, and selenoprotein S involved in immune responses [29]. Noteworthy, antioxidant activity, which helps to eliminate reactive oxygen species (ROS) and suppress oxidative stress-mediated cell damage, is exhibited by not only proteins from the GPx and TrxR groups but also other selenoproteins such as selenoproteins P, R, and W [30]. The synthesis of selenoproteins is modulated primarily by dietary Se intake, but there is a tissue-specific hierarchy of selenoprotein expression when Se stores are limited [31]. As demonstrated in rodent studies, the synthesis of GPx1 as well as selenoproteins W and H is suggested to be very sensitive to low Se conditions. Selenoprotein P and TrxR are moderately sensitive and DIO and GPx4 are the least affected by Se dietary status [32]. Very recently, a cell line-specific hierarchy of selenoproteins synthesis has been reported in an in vitro study [33]. In these investigations, enzymes GPx1 and GPx4 were mostly sensitive to Se concentration variations in HEK293, LNCaP, and HaCaT lines, while selenoproteins F, M, and S were most sensitive to Se conditions in the HepG2 cell line [33]. Single-nucleotide polymorphism in selenoprotein genes is suggested to modify the effect of dietary Se on selenoprotein activity [31] and influence the selenoprotein hierarchy [34].

As mentioned above, severe Se deficiency in animals and humans is directly linked with several diseases. In animals, very low Se status causes white muscle disease, i.e., a myodegenerative disorder in calves, lambs, kids, foals, and donkey foals. The disease involves degeneration of skeletal muscles as well as the diaphragm and the heart [35]. The clinical manifestations of this disorder are stiffness, weakness, trembling of the limbs, swollen muscles, and labored breathing [36]. Other diseases in animals that are related to Se deficiency include yellow fat disease (in horses and donkeys), which involves degeneration of adipose tissue that is replaced by connective tissue and calcium deposits, and VESD (vitamin E/Se deficiency) syndrome manifested by mulberry heart disease [35]. In humans, severe Se deficiency is associated with Keshan disease and Kashin–Beck disease [37]. Keshan disease is a cardiomyopathy (disease of the heart muscle) affecting mainly children aged 2–10 years and, to a lesser extent, women of childbearing age. The disease mainly occurs in regions of China with very low Se levels in soil. Se supplementation as well as foodstuff with higher Se content (not grown on local soils) has significantly prevented the incidence of Keshan disease in recent years. Besides the very low Se status, other agents like Coxsackie virus B4 infections and deficiencies of vitamin E, calcium, or molybdenum may also be involved in the etiology of Keshan disease [37, 38]. The second Se deficiency-related disorder is Kashin–Beck disease. This osteochondropathy (disease of the cartilage and bone) was reported in Tibet, China, Siberia, and North Korea [38]. Similar to Keshan disease, additional factors (besides Se deficiency) may also play a role in its development. These are fungal contamination of grain, humic substances in drinking water, iodine deficiency, and GPx1 gene polymorphism [37, 38]. In addition, studies revealed a decreased Se status in patients with chronic kidney disease [39], type 1 diabetes mellitus [40], epilepsy, cardiovascular diseases [20], and various cancer types [37]. Se supplementation was suggested to be beneficial in the treatment or prevention of the aforementioned disorders [37, 39]. Notably, the Se status is suggested to be critical in Se supplementation-mediated health effects. Accordingly, it was estimated that the beneficial effect of Se supplementation on human health should be largely seen in subjects with serum or plasma Se concentration less than 122 μg L^−1^. In contrast, Se administration to patients with a serum or plasma Se concentration of 122 μg L^−1^ or higher has been associated with an increased risk of nonmelanoma skin cancer and type 2 diabetes [20]. Additionally, as reported by Hesketh and Meplan [31], a functional single-nucleotide polymorphism in selenoprotein genes may be an important factor (besides environmental stress, lifestyle behavior, and Se intake) contributing to contradictory results in Se supplementation trials.

As highlighted in our previous review, Se has long been known for its ability to alleviate the deleterious effects of many toxic metals [10]. It is not surprising, therefore, that researchers still undertake studies to evaluate the therapeutic action of this mineral against metal toxicity. For example, very recently, Se has been shown to protect barium-induced heart injury in rats [41]. In another study, Se protected from chromium (CrVI)-induced damage in chicken brain. The supplementation of Se to these chickens prevented negative changes in the levels of reduced glutathione (GSH), mitochondrial membrane potential, and Ca^2+^-ATPase activity induced by Cr in brain tissue [42]. Protective effects of Se were also described against lithium-provoked oxidative stress in FaDu and Vero cell lines [43]. In chickens, simultaneous administration of Se with lead (Pb) reduced the Pb level in serum and alleviated Pb-induced activation of the NF-κB pathway [44]. Lastly, well known is the capability of Se to reduce the symptoms of mercury (Hg) toxicity in various experimental models such as mice [45], fish [46], and HepG2 cell culture [47].

Interestingly, before the discovery that Se is nutritionally essential, this element was mostly known as a poison for livestock and mammals [48]. Excessive dietary intake of Se causes Se poisoning, which was reported both in animals and humans. The symptoms generally involve dermatologic and neurologic problems. Chronic Se intoxication in livestock through ingestion of plants with high Se content (5–50 mg per kg of mass) is manifested as alkali disease, symptoms of which involve hoof deformities, lack of vitality, anemia, and stiffness [49]. Endemic human selenosis was described very thoroughly by studies of Se-excessive regions of China located in Western Hubei Province and Southern Shaanxi Province, where high Se content in soils, water, and corn was identified [37]. The main symptoms were loss of hair and nails, poor dental health, disorders of skin, and neurological disturbances. The dietary intake of Se in the regions affected by selenosis estimated in the 1970s was 3.2–6.8 mg day^−1^. Due to the lower dependence on locally grown food, no human cases of selenosis have been reported in those areas since 1987 [37]. During the last years, cases of selenosis in humans induced by environmental exposure to Se were reported in the state of Punjab, India, where the disease was caused by excessive Se content in locally grown grains [50]. Acute Se poisoning resulting in a massive alopecia was also described in a 55-year-old woman who ingested paradise nuts (*Lecythis ollaria*) [51]. Moreover, as reported by some epidemiologic studies, even low-level but chronic overexposure to Se, for example from drinking water, may be associated with adverse health effects in humans such as an increased risk of amyotrophic lateral sclerosis and cancer [52]. Therefore, in terms of the use of Se as a supplement against toxic metals (as described further in this article), the potential deleterious effects of chronic Se use on human health may be a challenge and an impediment to therapy (Table 1).

**Table 1:** List of abbreviations

Abbreviations	Chemical name	Abbreviations	Chemical name
Akt	Protein kinase B	iNOS	Inducible nitric oxide synthase
ALP	Alkaline phosphatase	JNK	c-Jun N-terminal kinase
ALT	Alanine aminotransferase	Keap1	Kelch-like ECH-associated protein 1
As	Arsenic	LDH	Lactate dehydrogenase
As^III^	Arsenite	LDL	Low density lipoprotein
As^V^	Arsenate	MDA	Malondialdehyde
AS3MT	Arsenite methyltransferase	MMA^III^	Methylarsonous acid
AST	Aspartate aminotransferase	MMA^V^	Methylarsonic acid
BW	Body weight	Na_2_SeO_3_	Sodium selenite
CAT	Catalase	Na_2_SeO_4_	Sodium selenate
Cd	Cadmium	NF-κB	Nuclear factor kappaB
COX-2	Cyclooxygenase-2	NO	Nitric oxide
DMA^III^	Dimethylarsinous acid	NQO1	NAD(P)H:quinone oxidoreductase 1
DMA^V^	Dimethylarsinic acid	Nrf2	Nuclear factor erythroid 2-related factor
ER	Endoplasmic reticulum	PI3K	Phosphatidylinositol 3-kinase
GCLC/M	Glutamate cysteine ligase catalytic/modifier subunit	PPAR-γ	Peroxisome proliferator-activated receptor
GPx	Glutathione peroxidase	ROS	Reactive oxygen species
GR	Glutathione reductase	SAM	S-adenosylmethionine
GSH	Reduced glutathione	SD rat	Sprague–Dawley rat
GSSG	Oxidized glutathione	Se	Selenium
GST	Glutathione S-transferase	SeMet	Selenomethionine
HB chicken	Hy-Line Brown chicken	SeNPs	Selenium nanoparticles
HDL	High density lipoprotein	SOD	Superoxide dismutase
HO-1	Heme oxygenase	TAC	Total antioxidant capacity
IB chicken	Isa Brown chicken	TNF-α	Tumor necrosis factor-α
IL	Interleukin	TrxR	Thioredoxin reductase

## Arsenic as a Toxicant

Arsenic, i.e., a naturally occurring nonmetal, exists in four oxidation states (+ 5, + 3, 0, − 3) in inorganic (arsenite, arsenate) and organic forms [53]. It is used in industry for a range of purposes such as electronics (e.g., semiconductor devices and high-power microwave devices) and manufacture of nonferrous alloys, glass, antifouling paints, and pharmaceutical substances [54]. In addition, four organoarsenic drugs, namely roxarsone, carbasone, arsanilic acid, and nitarsone, were used for many years as veterinary feed additives to prevent certain diseases in poultry. However, in 2014 and 2015, the U.S. Food and Drug Administration formally withdrew the approval for the use of these animal drug products [55]. Arsenic present in inorganic species, i.e., arsenite (+ 3 oxidation state of As, As^III^) and arsenate (+ 5 oxidation state of As, As^V^), is considered a health-threatening environmental pollutant due to its high toxicity potential and frequent occurrence in food and water at levels that pose a health risk for millions of people [56]. High concentration of As in groundwater was reported mainly in South and East Asia, with Bangladesh and West Bengal of India as the most polluted areas. In these regions, As water pollution derives from natural geological sources [56, 57]. However, anthropogenic activities, such as burning As-rich coal [58] and As-based pesticides [59], can also contribute to As contamination of the environment and pose a health hazard to humans. Furthermore, a high concentration of organic As compounds is found in many kinds of seafood [60]. These are nontoxic arsenobetaine (finfish and shellfish) and potentially toxic arsenosugars (seaweed) and arsenolipids (fish oils). As recently reviewed by Taylor et al. [60], the heath risk posed by seafood-derived As still needs evaluation due to the lack of data on its toxicity and chronic exposure in humans and other mammals.

Arsenic poisoning produces multiple harmful health effects in humans. Acute As toxicity is most often caused by accidental ingestion of pesticides or in attempted suicide. The clinical features of acute As poisoning include abdominal pain, vomiting, diarrhea, skin rash, and toxic cardiomyopathy. Toxic manifestations of chronic As exposure vary between individual people, population groups, and geographic areas [57]. The major symptoms involve skin problems (hyperpigmentation, palmar, and solar keratosis), skin cancer, internal cancers (bladder, kidney, lung), diseases of the blood vessels of the legs and feet, and possibly diabetes mellitus and hypertension [56, 57]. The latest evaluation by the IARC Committee has considered As and inorganic As to be carcinogenic to humans (group 1), causing cancer of the lungs, urinary bladder, and skin [54]. The basic mechanisms underlying As toxicity include generation of ROS, inhibition of various enzymes such as those involved in GSH production, interference with gene expression of proteins, and promotion of production of inflammatory mediators [61]. Oxidative DNA damage and inhibition of DNA repair are the main underlying mechanisms of As genotoxicity [62]. Other proposed mechanisms include changes in DNA methylation, aneuploidy, and gene amplification [54].

Epidemiologic data show that humans exposed to the same levels of As exhibit individual differences in the susceptibility to As-related disorders. Polymorphism of genes coding for enzymes involved in As metabolism (e.g., arsenite methyltransferase), detoxification enzymes (glutathione S-transferase), or DNA repair proteins is mentioned as a major genetic factor associated with interindividual variability in response to As exposure [63]. Other factors such as age [64], smoking, and use of fertilizers [65] and alcohol [66] may also significantly modify As metabolism and toxicity. In addition, nutritional factors are also indicated to affect individual susceptibility to As [67]. Accordingly, daily diet deficient in protein and micronutrients increases the vulnerability to As toxicity. Nutrients such as vitamins, minerals, protein, carotenoids, etc. enhance detoxification of As by enhancing methylation of As and antioxidative mechanisms [67].

## Cadmium as a Toxicant

Cd is a heavy metal belonging to group 12 of the periodic table. It exists predominantly in the + 2 oxidation state and does not undergo oxidation–reduction reactions [68]. Cd has various industrial applications such as production of nickel–cadmium batteries, pigments, protective plating of steel, plastic stabilizers, and alloys. However, due to its high toxicity, the use of Cd for pigments, stabilizers, and coatings has been restricted, particularly in the European Union [69]. Cd emissions to the environment originate from natural and anthropogenic sources. Natural Cd emissions include volcanic activity, weathering of Cd-containing rocks, and forest fires. Emissions of Cd related to human activities include nonferrous metal production, combustion of fossil fuels, waste incineration (especially Cd-containing batteries and plastics), and manufacture of phosphate fertilizers [70, 71]. For the general population, the primary environmental source of Cd exposure is food with average daily intake of 0.1–0.4 μg Cd kg^−1^ BW in most countries. Smoking is a significant source of Cd exposure for smokers. The major route of occupational exposure to Cd is through inhalation of dust and fumes [69].

The most sensitive toxicological endpoints of Cd are the kidney and bone following oral exposure and the kidney and lung following inhalation exposure [68]. Cd and its compounds have been classified in group 1 of carcinogens in an IARC monograph published in 2012 [69]. This classification was based on several epidemiologic studies performed between 1976 and 2004 and showing increased lung cancer risks among workers occupationally exposed to cadmium and on a prospective population-based study performed by Nawrot et al. [72] showing increased cancer risk in inhabitants of a Cd-polluted area in Belgium [69]. The most severe form of chronic Cd poisoning is known as “itai-itai” disease reported in the Cd-polluted Jinzu River basin of Toyoma, Japan [73, 74]. The disease process is characterized by renal tubular dysfunction, osteomalacia, osteoporosis, bone pain, and waddling gait leading to disabling condition. The source of the Cd exposure was Cd-contaminated rice and drinking water polluted by Cd discharge from a mine located upstream of the Jinzu River. After official recognition by the government (in 1968) that the Cd exposure was the cause of the itai-itai disease cases, efforts were undertaken to reduce Cd pollution in that region. The intervention program (1980–2012) involved soil replacement in paddy fields, and since then, the Cd levels in rice grains have significantly declined [73, 74]. However, due to accumulation of Cd in the kidneys and its long biological half life promoted by binding to metallothioneins [75], new cases of itai-itai diseases are being diagnosed even today [73, 74]. Furthermore, as reported by most recent epidemiologic studies, low environmental Cd exposures are also linked with adverse health effects in humans. These include renal tubular disorders [76], osteoporosis in postmenopausal women [77], cardiovascular diseases [78], and psoriasis [79].

Central events in Cd-induced tissue injury include inflammation and apoptotic cell death through the mitochondrial and ER-mediated pathways and the p53-dependent pathway [80, 81]. In addition, Cd excess was demonstrated to promote autophagy in tissues by modulating NF-κB/JNK [82] and ER stress/IRE-1/JNK [83] signaling pathways. Cd exposure causes the generation of ROS, genotoxicity, and impairment of cell growth signaling, as described below. As a redox inert metal, Cd itself cannot induce ROS production in a direct manner. Still, Cd can inhibit the activity of antioxidant enzymes such as Cu/Zn SOD and CAT (probably through direct Cd/enzyme interaction), which decreases intracellular antioxidant defenses [84]. Cd was also shown to replace iron and copper in metalloproteins like ferritin, leading to elevation of toxic-free iron and copper [85], and deplete GSH levels through Cd–glutathione complex formation and/or oxidation of GSH to its oxidized form [86]. Lastly, Cd interferes with the mitochondrial electron transport chain, specifically complex II and complex III, and inhibits their activity [87]. All these perturbations eventually raise ROS levels in cells, leading to oxidative stress. Furthermore, Cd can also evoke damage to DNA mainly through indirect mechanisms involving induction of oxidative stress and inactivation of DNA repair proteins such as human 8-oxoguanine-DNA-glycosylase (enzyme responsible for repair of 7,8-dihydro-8-oxyguanine, oxidative DNA lesion) [69]. Lastly, it was also suggested that Cd-induced epigenetic changes such as alteration in DNA methylation can contribute to Cd-associated disorders, including tumor promotion [69, 88].

## Use of Se in the Prevention of As Intoxication in Animal and Cell Culture Models

For the purpose of this chapter, a systemic search was performed on PubMed with the terms “selenium” and “arsenic” in combination with “protective,” “antioxidant,” “toxicity,” and “toxic.” I limited the search to studies published in 2012–2018 (earlier studies were reviewed by us previously). In this chapter, the focus was placed solely on animal-based experimental studies (in vivo and in vitro models) that tested Se supplementation as a potential antioxidant/antidote against As toxicity (recent human studies of As and Se interactive effects will be described further in this review).

The search of the literature from the reviewed period (2012–2018) showed 12 experimental studies investigating the impact of Se supplementation on the toxicity of As^III^ in animal and cell culture models (Table 2). Several of these studies conducted in a rat model have shown the protective ability of Se (as selenite or Se from lentils) against arsenite-induced hepatic oxidative damage [90–92, 96]. For example, 20-week selenite administration (17 mg L^−1^ in drinking water, selenite intakes 1.61–2.49 mg kg^−1^ BW day^−1^) to rats that were concurrently exposed to arsenite (13 mg L^−1^ in drinking water, arsenite intakes 1.24–1.90 mg kg^−1^ BW day^−1^) significantly reduced lipid peroxidation and restored GPx activity in the liver [92]. In this study, the Se treatment also partly improved the expression of antioxidant-related genes encoding SOD1, CAT, TrxR, and GPx, which were downregulated in the As-treated group. Simultaneously, the levels of oxidative stress proteins, HSP70 and HO-1 (indicators of cellular stress), were sharply increased in the As group, and Se administration attenuated this increase. The authors concluded that, through adjusting the expression of oxidative stress-related genes, Se improved the activities of antioxidant enzymes, which in turn protected liver cells from As-induced oxidative damage [92]. In line, other reports showed that oral selenite supplementation (3 mg kg^−1^ BW) notably mitigated signs of arsenite (5.5 mg kg^−1^ BW) hepatic toxicity such as lipid peroxidation [91], decrease in GSH levels [90], and arsenite-mediated damage in the liver architecture [90, 91].

**Table 2:** Summary of studies evaluating Se effects on As^III^-induced toxicity in animal and cell culture models

Duration of Se treatment	Se type	Se dose	Model	Main target	Effects of Se on the toxicity of As^III^	References
6–14 days	Na_2_SeO_3_	0.025 mg Se kg^−1^ BW oral (drinking water)	Pregnant Syrian hamster	Fetus	↓ As content in the brain, liver, kidney, bladder, and skin of pregnant animals; ↓ As accumulation in placenta and whole fetuses; ↓ primary and ↑ secondary methylation index in urine and tissues of dams and in whole fetuses; ↓ activities of GR, SOD1, and CAT, which were increased by As; further increase in the activity of GPx, which was already increased by As; ↑ viable fetuses and ↓ nonviable fetuses and fetal resorptions; partly prevented As-mediated body weight loss in pups	[89]
3 weeks	Na_2_SeO_3_	3 mg kg^−1^ BW oral intubation	Wistar rat	Liver	↓ AST, ALT, and ALP activities in plasma compared to As-treated animals; ↑ GSH level and GPx activity, ↓ lipid peroxidation and GST activity; ↓ As-induced histological changes such as cytoplasmic vacuolization	[90]
3 weeks	Na_2_SeO_3_	3 mg kg^−1^ BW oral intubation	SD rat	Liver	Partly protected against an As-induced increase in liver weights; ↓ AST and ALT activities in serum, which were increased by As; ↓ levels of MDA, NO, advanced oxidation protein products, and serum IL-6, which were elevated by As; ↑ TrxR and TAC activities; ↑ mRNA gene expression of Nrf2, which was decreased by As; ↓ As-mediated histopathological changes such as inflammatory cellular infiltration	[91]
20 weeks	Na_2_SeO_3_	17.0 mg L^−1^ oral	SD rat	Liver	↓ ALT and AST activities in the blood, which were increased by As; ↓ lipid peroxidation; ↑ GPx activity, which was reduced by As; ↑ mRNA expression of GPx, CAT, SOD1, Txnrd1, and protein expression of TrxR, which were reduced by As; ↓ As-induced HSP70 and HO-1 protein expression	[92]
50 days	SeMet	2 ppm oral (in diet)	C57BL/6 N mouse	Liver	Se did not have an effect on As excretion in urine; ↑ lipid peroxidation in the liver of Se-only–treated mice and As-treated mice	[93]
2 h	Na_2_SeO_3_	1, 5, and 10 μM	*Poeciliopsis lucida hepatocellular* carcinoma *cell* line (*PLHC*-*1*)		↓ As-induced cytotoxicity and the ROS level after 10-h As exposure; ↑ GPx activity, which was reduced by As; partly protected from mitochondrial membrane potential damage induced after 10 and 20 h As exposure; ↓ As-mediated apoptosis after short As exposure (10 h) but ↑ As-mediated apoptosis in the case of longer As exposure (40 h)	[94]
13 weeks	Not specified (Se-rich lentils)	0.009 (Se-deficient), 0.16 (Se-adequate), 0.3 mg Se kg^−1^ (Se-high) oral	ApoE^−/−^ mouse	Heart, liver	Se-high diet reduced or prevented atherosclerotic plaque formation in the aortic sinus and aortic arches, respectively, compared to Se-deficient and Se-adequate diets; Se-adequate and Se-high diet increased the HDL:LDL ratio, which was decreased by As; Se-adequate and Se-high diet decreased GSH levels and increased the GSSG level in the livers of As-treated mice, compared to Se-deficient diet.	[95]
14 weeks	Not specified (Se-rich lentils)	< 0.01 ppm Se (Se-deficient), 0.3 ppm Se (Se-high) oral	Wistar rat	Blood, kidney, liver	Se-high diet reduced As levels in kidney but increased As levels in urine, and feces, compared to Se-deficient diet; ↑ GSH levels in blood, which were reduced in Se-deficient diet; mitigated hepatic lipid peroxidation; partly recovered antibody response, which was reduced in Se-deficient animals	[96]
6 weeks	Na_2_SeO_3_	3 mg/kg BW oral intubation	SD rat	Kidney, heart	↓ plasma renal markers (urea, creatinine, blood urea nitrogen), which were increased by As; ↓ As-induced lipid peroxidation in the kidney and heart; ↑ kidney and heart levels of GSH, SOD, and CAT, which were decreased by As; ↓ cardiac risk factors such as plasma triglyceride levels, which were increased by As; ↓ histopathological changes in renal tissue	[97]
24 h	SeMet	100 μM	*Human* embryonic *kidney cell line* (*HEK-293*)		↓ As-induced cytotoxicity and the ROS level; enhanced phosphorylation of proteins involved in ROS detoxification, antitumor activity, and cell growth	[98]
1 h	SeNPs	0.01 μg μL^−1^	Human lymphocytes		Nanoselenium prevented As-induced cytotoxicity and DNA damage	[99]
48 h	Na_2_SeO_3_	10 μM	*Rat pheochromocytoma cell line* (*PC12*)		↓ As content; ↓ As-induced LDH leakage to the culture medium; ↑ GSH levels and GPx activity, which were reduced by As; ↓ lipid peroxidation; ↑ expression of proteins involved in inhibition of autophagy (mTOR, Akt), which were downregulated by As; ↓ As-induced upregulation of proteins that induce autophagy (p62, ubiquitin); ↑ expression of antiapoptotic proteins (bcl2, NF-κB, ERK1) and ↓ proapoptotic proteins (Bax, caspase-3); ↓ mRNA expression of caspase-9, which was increased by As	[100]

*↑*, increased; *↓*, decreased

Additionally, other recent studies reported the protective capability of Se (from selenite or lentils) against arsenite-induced renal toxicity [97], cardiovascular damage [95, 97], or immunotoxicity [96]. For example, in arsenite-intoxicated rats (10 mg kg^−1^ BW day^−1^), oral selenite treatment (3 mg kg^−1^ BW day^−1^) inhibited renal lipid peroxidation as well as toxic effects of arsenite on antioxidant enzymes (SOD and CAT) and prevented arsenite-mediated histopathological changes in kidney tissue [97]. In a hamster model, Sampayo-Reyes et al. [89] used selenite to elucidate its effectiveness in the suppression of arsenite-induced teratogenic changes. They found that selenite given orally (0.025 mg Se kg^−1^ BW day^−1^ in drinking water) to female hamsters from the 1st or 8th day of gestation to the time of delivery (on days 13–14) was efficacious in the prevention of teratogenic effects induced by arsenite (100 ppm in drinking water) [89]. In this study, selenite supplementation reduced the number of nonviable fetuses and fetal resorptions and increased the number of viable fetuses in arsenite-treated hamsters. Selenite also improved body weight gain of in utero arsenite-exposed pups measured at postnatal days 20 and 30, compared to arsenite-only–treated hamsters. Further results showed reduced As accumulation in dams’ tissues and whole fetuses and changes in As methylation indexes (a decrease in the primary methylation index and an increase in the secondary methylation index in urine and tissues of dams and in fetuses), which along with the increased antioxidant potential were indicated as the main mechanisms underlying selenite-mediated protection against arsenite-induced teratogenic effects [89].

Several recent cell-based studies have confirmed that various Se forms have the potential to counteract arsenite-triggered toxicity. For instance, Prasad and Selvaraj [99] demonstrated that Se nanoparticles (0.01 μg μL^−1^) mitigated arsenite (10 μM)-induced cell death in cultured human lymphocytes. Using the comet assay, the researchers also found that nanoSe prevented arsenite-dependent DNA damage [99]. In another in vitro study, an organic Se form (SeMet, 100 μM) protected human kidney cells from arsenite (30 μM)-induced cell cytotoxicity and ROS generation [98]. Inorganic Se administered as selenite (10 μΜ) induced antioxidant, antiautophagic, and antiapoptotic effects in arsenite (10 μΜ)-treated PC12 cells [100]. Finally, selenite (1, 5, and 10 μM) pretreatment of PLHC-1 cells reduced arsenic trioxide (100 μM)-induced toxic effects such as ROS generation, DNA damage, and apoptosis [94]. In this study, however, the protective effects of selenite were observed principally at a short time of As^III^ exposure (10 h), whereas toxicity induced by longer As^III^ treatment (40 h) was rather increased by selenite.

**Fig. 1:**
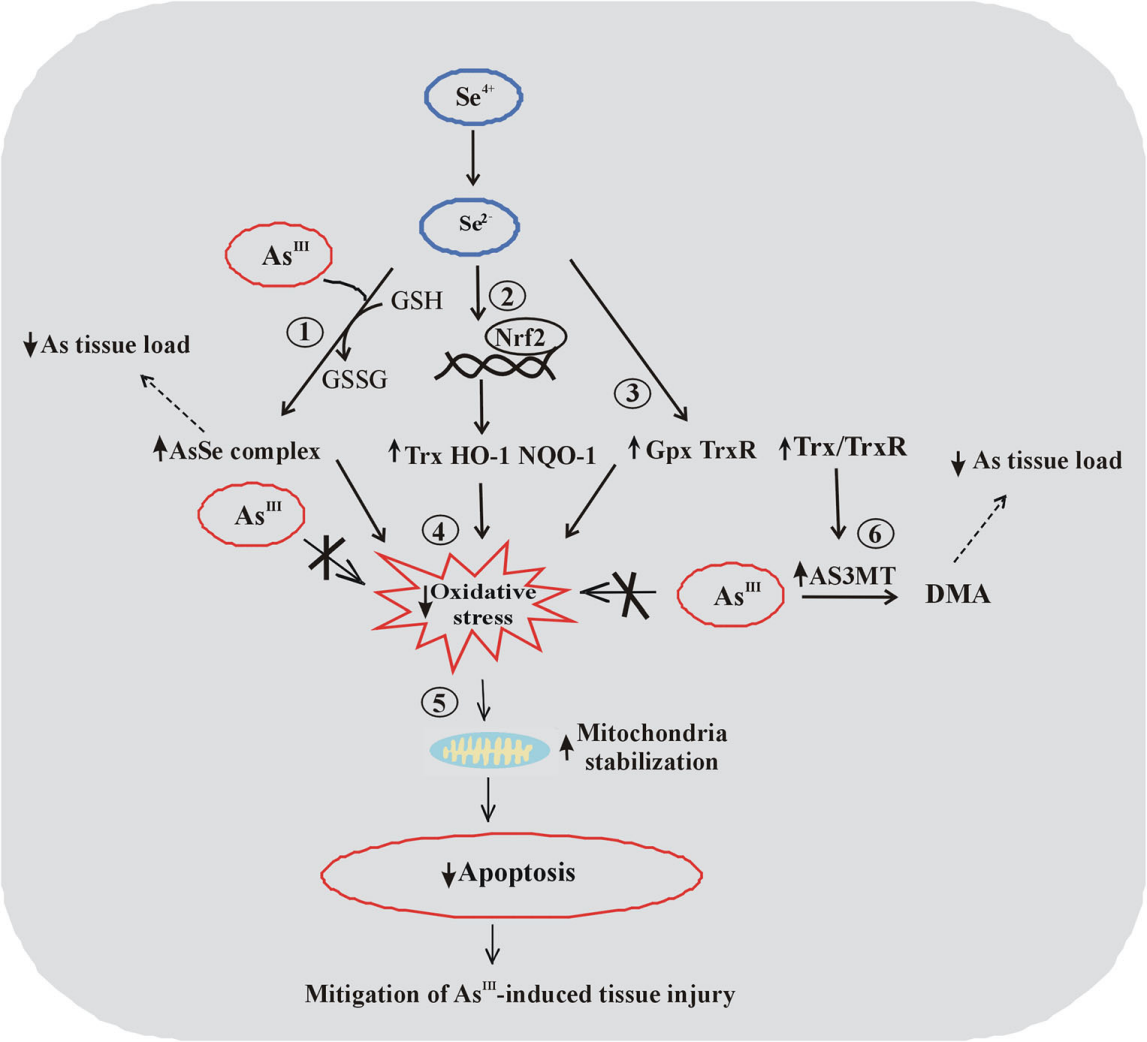
Schematic representation of possible Se effects against As^III^-induced intoxication observed in recent studies. The three following basic Se effects (1) inhibition of As accumulation via formation of As–Se complexes, (2) activation of the Nrf2 factor, and (3) stimulation of GPx and TrxR activity most probably contribute to (4) a decrease in As-induced oxidative stress generation followed by (5) stabilization of mitochondria and blockade of As-induced apoptosis. In addition, stimulation of TrxR activity may contribute to (6) enhancement of As methylation capacity catalyzed by AS3MT. ↑, increased; ↓, decreased

Possible biological pathways that can be involved in mediation of Se effects against As^III^ toxicity are illustrated in Fig. 1.

## Use of Se in the Prevention of Cd Intoxication in Animal and Cell Culture Models

Articles for this section were searched in PubMed using the keywords “selenium” and “cadmium” in combination with “protective,” “antioxidant,” “toxicity,” and “toxic.” Similar to the previous chapter, 2012 was used as a starting point and the search for articles was limited to animal-based experimental studies (in vivo and in vitro models) that tested Se supplementation as a potential antioxidant/antidote against Cd toxicity.

The search of databases yielded 18 experimental reports that were the basis for writing of this chapter (Table 3). The articles were devoted to elucidation of potential protective mechanisms of Se (usually in the form of selenite) against, e.g., Cd-induced nephrotoxicity [101, 103], hepatotoxicity [104, 105], immunotoxicity [106], reproductive toxicity [112, 113], and neurotoxicity [114, 115]. With respect to the renal protective effects of Se, Liu et al. [102] reported that selenite added at supranutritional dose (10 mg kg^−1^ diet) to birds’ feed mitigated CdCl_2_ (150 mg kg^−1^ diet)-induced apoptosis of kidney cells through decreased Cd accumulation and amelioration of oxidative stress and ER stress. Moreover, a recent investigation by Bao et al. [101] was designed to find out if the phosphatidylinositol 3-kinase (PI3K)/protein kinase B (Akt) pathway, which maintains cell survival and prevents apoptosis, may be related to Se and Cd effects on chicken kidneys. The study demonstrated that 2 mg Se kg^−1^ diet (as selenite) can restore the PI3K/Akt signaling pathway impaired by CdCl_2_ (150 mg kg^−1^ diet) and thus reduce toxic effects of Cd on mitochondria and induction of apoptosis [101]. Se also exerted effects against hepatotoxicity induced by Cd in birds [104]. The results showed that selenite (10 mg kg^−1^ diet) decreased Cd accumulation and enhanced antioxidant defense, which attenuated Cd-mediated adverse morphological changes and oxidative stress in hepatic tissue [104]. Other researchers investigated the influence of Se on Cd toxicity in chicken pancreas [111]. They have found that Se can attenuate Cd-induced apoptosis of pancreatic cells through regulation of the PPAR-γ/PI3K/Akt pathway.

**Table 3:** Summary of studies evaluating Se effects on Cd-induced toxicity in animal and cell culture models

Duration of Se treatment	Se type	Se dose	Model	Main target	Effects of Se on the toxicity of Cd	References
90 days	Na_2_SeO_3_	2 mg Se kg^−1^ diet	HB chicken	Kidney	↓ Cd content; ↓ NO level, iNOS activity and apoptosis, which were increased by Cd; ↑ activities of ATPase and mitochondrial respiratory chain complexes, which were decreased by Cd; ↓ mRNA and protein expression of apoptosis-related genes (Bax, Bak, p53, caspase-3, caspase-9, cytochrome c, which were induced by Cd; ↑ mRNA expression of PI3K and Akt, which was reduced by Cd	[101]
90 days	Na_2_SeO_3_	2 mg kg^−1^ diet	HB chicken	Kidney	↓ uric acid and urea nitrogen level in serum, which were increased by Cd; ↑ mRNA expression of miR-30a, which was reduced by Cd; ↓ expression of ER stress-related genes (GRP78 and IRE-1) and autophagy-related genes (Beclin-1, LC3-I, LC3-II, and ATG5), which were increased by Cd; ↓ Cd-mediated ultrastructural changes	[83]
60 days	Na_2_SeO_3_	10 mg kg^−1^ diet	IB chicken	Kidney	↓ Cd content; ↓ MDA and NO level, iNOS activity, and number of apoptotic cells, which were increased by Cd; ↑ SOD and GPx activities, which were reduced by Cd; ↓ mRNA expression of ER stress-related genes (e.g., GRP78 and GRP94), which were increased by Cd; partial restoration of Cd-induced changes in mRNA expression of apoptosis-related genes (bcl-2 and caspase-3); ↓ Cd-mediated histopathological and ultrastructural changes	[102]
30 days	Na_2_SeO_3_	0.1 mg Se kg^−1^ BW oral (drinking water)	Albino rat	Blood, liver, kidney	↓ Cd content in the liver; ↓ TNF-α, IL-6, and IL10 in serum, which were elevated by Cd; ↑ activities of GPx, CAT, and SOD and ↑ GSH levels in serum, which were reduced by Cd; protection against lipid peroxidation in serum; improvement of serum hepatic and renal markers	[103]
60 days	Na_2_SeO_3_	10 mg kg^−1^ diet	IB chicken	Liver	↓ Cd content; ↓ number of apoptotic cells, lipid peroxidation, NO level, and total NOS activity, which were increased by Cd; ↑ SOD and GPx activity, which were decreased by Cd; ↓ Cd-dependent histopathological and ultrastructural changes;	[104]
24 h	Na_2_SeO_3_	1 μM	Chicken hepatocytes		↓ Cd-induced morphological damage to heptocytes; ↓ Cd-related elevation of ALT and AST activities and LDH release; ↓ ROS and MDA contents, which were increased by Cd; ↑ GPx activity and T-AOC content; ↓ Cd-mediated apoptosis and autophagy induction; ↑ mRNA expression of Nrf2 and Nrf2-related genes (NQO1, HO-1, GST, GCLC/M), which were downregulated by Cd.	[105]
90 days	Na_2_SeO_3_	2 mg Se kg^−1^ diet	HB chicken	Spleen	↓ Cd content; ↑ activity of SOD, CAT, and GPx, which were reduced by Cd; ↓ Cd-induced MDA and H_2_O_2_ content; ↑ mRNA and protein expression of GPx-1 and TrxR1; ↓ number of apoptotic cells and histopathological changes, which were increased by Cd; ↓ Cd-mediated Nrf2 nuclear accumulation and mRNA expression of Nrf2-related genes (HO-1 and NQO1)	[106]
84 days	Na_2_SeO_3_	2 mg Se kg^−1^ diet	IB chicken	Neutrophils	↓ mRNA expression of NF-κB and COX-2, which were induced by Cd; ↑ mRNA expression of iNOS, compared to the Cd-treated group; ↓ Cd-induced mRNA expression of HSP40 and HSP70	[107]
12–48 h	Na_2_SeO_3_	100 nM	Chicken neutrophils		↓ iNOS activity and NO level, which were increased by Cd; ↓ number of apoptotic cells; ↓ mRNA expression of Bak and ER stress-related genes (GRP78 and ATF6), which was induced by Cd; ↓ mRNA expression of IL-1β, IL-10, iNOS, PGE_2_, IL-4, NF-κB, COX-2, and TNF-α; ↓ protein expression of NF-κB and caspase-12	[108]
12–48 h	Na_2_SeO_3_	100 nM	Chicken lymphocytes		↑ GPx, SOD, and CAT activities, which were decreased by Cd; ↓ Cd-induced ROS and MDA levels; ↓ number of apoptotic cells, which was increased by Cd; ↓ mRNA expression of Bak, cytochrome c, p53, caspase-3, and caspase-9; ↑ mRNA expression of Bcl-2, Bcl-x, CaM; partial restoration of the intracellular level of Ca^2+^, which was increased by Cd	[109]
12–60 h	Na_2_SeO_3_	100 nM	Chicken lymphocytes		↓ iNOS activity and NO levels, which were increased by Cd; ↓ mRNA expression of NF-κB, iNOS, COX-2, TNF-α, and PGE_2_ genes, which was induced by Cd; ↓ protein expression of NF-κB and COX-2	[81]
90 days	Na_2_SeO_3_	2 mg Se kg^−1^ diet	HB chicken	Pancreas	↑ SOD, CAT, GPx, and total antioxidant capacity, which were decreased by Cd; ↓ mRNA expression of autophagy-related genes (e.g., dynein, Beclin 1) induced by Cd	[110]
90 days	Na_2_SeO_3_	2 mg Se kg^−1^ diet	HB chicken	Pancreas	↑ lipase, trypsin, and amylase activities which were reduced by Cd; ↓ NO level, iNOS activity, and number of apoptotic cells which were increased by Cd; ↓ expression of apoptosis-related genes (Bax, cytochrome c, and caspase-3) which were induced by Cd; ↑ expression of PPAR-γ/PI3K/Akt pathway-related genes which were reduced by Cd; ↓Cd-mediated ultrastructural changes	[111]
15, 25, 35 days	Na_2_SeO_3_	0.1, 0.2, 0.4 Se mg kg^−1^ BW oral by gavage	ICR mouse	Testes	Protection against Cd-induced deficits in sperm parameters (concentration, motility, and morphology); ↑ serum testosterone levels, which were reduced by Cd; upregulation of the expression of steroidogenic acute regulatory (StAR) protein and 17β-hydroxysteroid dehydrogenase (17β-HSD), which was decreased by Cd	[112]
84 days	Na_2_SeO_3_	2 mg Se kg^−1^ diet	IB chicken	Ovary	↓ Cd content; ↑ estradiol and progesterone levels; ↑ SOD and GPx activities, which were reduced by Cd; ↓ lipid peroxidation, NO and iNOS activity; ↓ mRNA and protein expression of ER stress-related genes (GRP78, ATF4, ATF6, IRE) and the caspase-3 gene, which were induced by Cd; ↓ Cd-dependent ultrastructural changes	[113]
60 days	Na_2_SeO_3_	10 mg kg^−1^ diet	IB chicken	Brain	↓ Cd content; ↓ NO level, mRNA expression and activity of iNOS, which were increased by Cd; ↓ Cd-mediated oxidative stress, ultrastructural and histopathological damage	[114]
24 h	Na_2_SeO_3_	100 nM	*Human neuroblastoma SH*-*SY5Y cell line*		↑ cell viability, which was decreased by Cd; ↓ Cd-induced ROS generation; ↓ GRP78 expression, which was upregulated by Cd; prevention of Cd-mediated Bax expression, caspase-3 activity, cytochrome c release, and GAP-43 downregulation	[115]
16 weeks	Na_2_SeO_4_	1.6 ppm Se oral (drinking water)	C57BL/6J mouse	Lung	↓ Cd content; Se prevented Cd-induced changes in transcripts for inflammation and myogenesis pathways and diminished Cd effects on other pathways such as coagulation and complement activation; Se alleviated Cd-disrupted metabolic pathways in amino acid metabolism and urea cycle	[116]

*↑*, increase; *↓*, decrease

Many studies have focused on the role of Se in the treatment of Cd-induced immunotoxicity. For example, dietary administration of selenite (2 mg kg^−1^ diet) to chickens treated with CdCl_2_ (150 mg kg^−1^ diet) reduced Cd-induced oxidative stress and detrimental Cd effects on the activities of antioxidant enzymes (GPx, CAT, SOD) in the spleen [106]. The protective activity of Se against proinflammatory effects of Cd in chickens was demonstrated by Tan et al. [107]. The study showed that selenite (2 mg kg^−1^ diet) decreased the levels of expression of proinflammatory factors (NF-κB and COX-2) that were upregulated in peripheral blood neutrophils following Cd exposure [107]. In addition, some in vitro studies examined the molecular mechanism of the protective Se effects against Cd-induced apoptosis of chicken neutrophils [108] and lymphocytes [109]. The results of these investigations revealed that selenite (100 nM) ameliorated apoptosis triggered by 1 μM CdCl_2_ exposure via the endoplasmic reticulum [108] and mitochondria-dependent pathways [109].

In the case of Cd-mediated reproductive damage, Ren et al. [112] observed that oral selenite pretreatment (0.1–0.4 mg kg^−1^ BW, by gavage) of mice intoxicated with CdCl_2_ (5 mg kg^−1^ BW) improved serum testosterone levels, sperm morphology and count, and sperm motility compared to the CdCl_2_-exposed group. In addition, Se enhanced the expression of steroidogenic acute regulatory (StAR) protein and testosterone synthetic enzymes, which were reduced following the Cd exposure. It was concluded that the protective effect of Se on Cd-mediated testicular impairments was due to an increase in the testosterone level via stimulation of StAR and testosterone synthetic enzyme activity [112]. Another study investigated the effectiveness of Se-enriched diet (2 mg Se kg^−1^ diet) on bird female reproductive impairments caused by exposure to CdCl_2_ (150 mg kg^−1^ diet) [113]. In this report, the Se treatment decreased Cd accumulation in the ovary and increased estrogen and progesterone contents. Additionally, Se inhibited Cd-induced apoptosis of ovarian cells via reducing oxidative stress-mediated ER stress pathway induction [113].

Molecular mechanisms involved in protective Se effects against Cd-mediated neurotoxicity were investigated using an undifferentiated SH-SY5Y human neuroblastoma cell line [115]. The results demonstrated that pretreatment of undifferentiated SH-SY5Y cells with selenite (100 nM) followed by CdCl_2_ (10 μM) exposure reduced Cd-dependent ROS generation and activation of ER stress and mitochondrial apoptotic pathways. The beneficial influence of Se included also an increase in GAP43 (growth associated protein 43) expression levels, a protein involved in neurite outgrowth, whose level was significantly diminished by Cd. Nevertheless, no protective effects of selenite were observed in differentiated SH-SY5Y cells, which proves that the neuroprotection offered by Se is dependent on the neuronal phenotype [115].

**Fig. 2:**
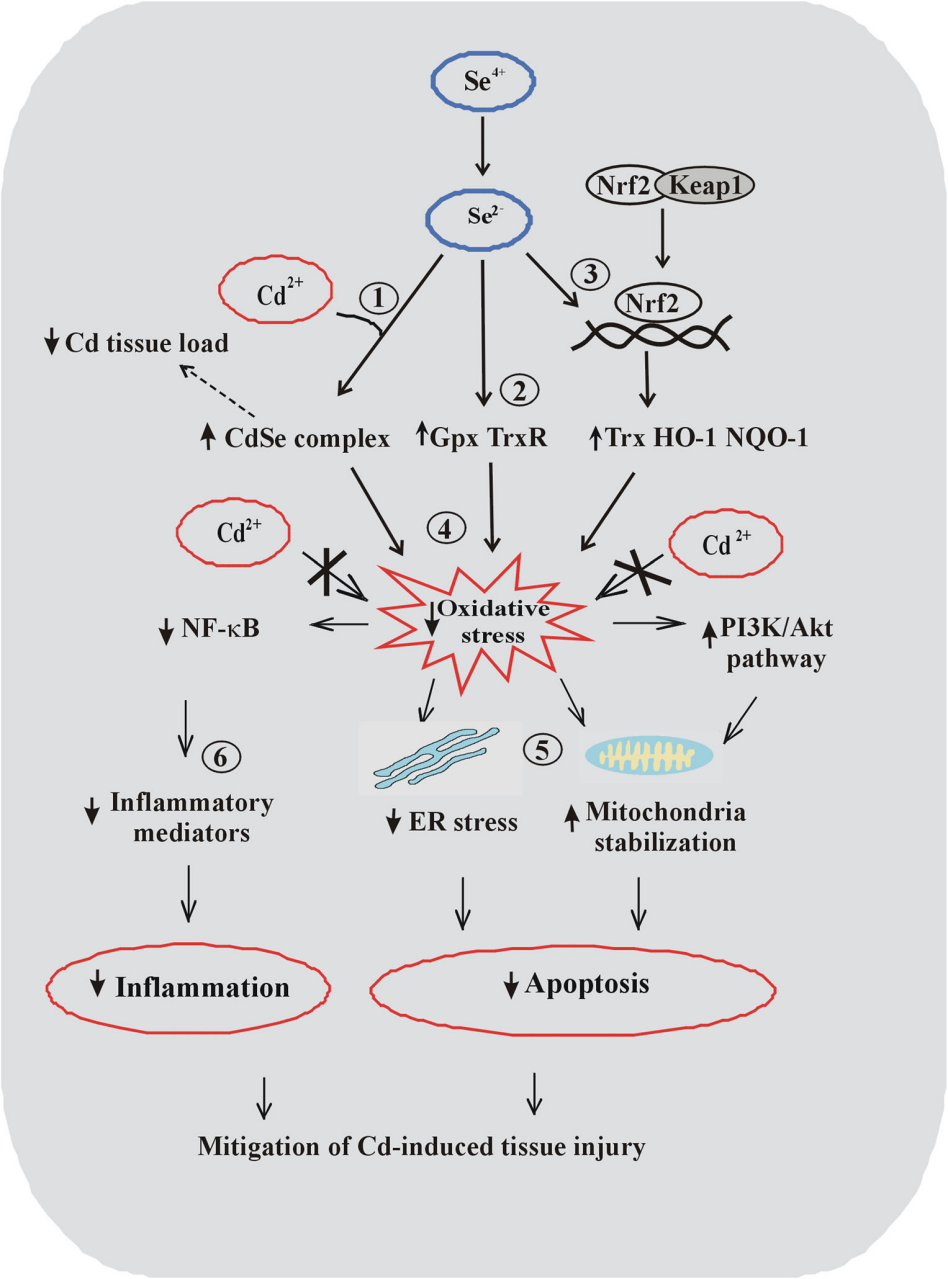
Schematic representation of possible Se effects against Cd-induced intoxication observed in recent animal and cell culture models. The three following basic Se effects (1) inhibition of Cd accumulation via formation of Cd–Se complexes, (2) stimulation of GPx and TrxR activity, and (3) activation of the Nrf2 factor most probably contribute to (4) a decrease in Cd-induced oxidative stress generation followed by (5) stabilization of mitochondria and ER and blockade of Cd-induced apoptosis and (6) decreased synthesis of inflammatory mediators, which in turn reduces Cd-mediated inflammation. ↑, increased; ↓, decreased

Considering the most recent animal and cell culture studies cited above, independent and overlapping biological pathways may be involved in the protective Se effects against Cd toxicity. The general summary of these pathways is depicted in Fig. 2.

## Mechanisms of Se Protection Against As and Cd Toxicity

As described in the previous chapters, a number of studies over the past few years have shown that Se supplementation exerted a protective effect against arsenite- or cadmium-induced toxicity in the liver (e.g., [91, 104]), kidney (e.g., [96, 102]), lung [116], or fetuses [89] in animal models and in cell cultures (e.g., [94, 98, 109]). These reports support the notion that the Se-mediated mechanism of As^III^ and Cd detoxification mostly relies on decreasing the As and Cd burden in tissues and improved the activity of antioxidant enzymes, as described below.

## Formation of As–Se and Cd–Se Compounds

Se-mediated reduction of As content in tissues is rationalized by the formation of a nontoxic As–Se conjugate, namely a seleno-bis(S-glutathionyl) arsinium ion [(GS)_2_AsSe]^−^. The compound has been identified in the bile of experimental rabbits [117] and rats [118] following exposure to selenite (or selenate) and arsenite. The precise mechanism of [(GS)_2_AsSe]^−^ generation has been thoroughly described in review articles by Gailer [119] and Sun et al. [62]. Accordingly, hydrogen selenide (HSe^−^ produced from selenite in the presence of GSH) reacts inside erythrocytes and probably hepatocytes with (GS)_2_As-OH (produced in the reaction of arsenite and GSH), forming a nontoxic complex seleno-bis(S-glutathionyl) arsinium ion [(GS)_2_AsSe]^−^. Once formed, the [(GS)_2_AsSe]^−^ species are removed out of the cells followed by rapid excretion via the liver and bile into the intestinal tract [120]. The complex [(GS)_2_AsSe]^−^ is proposed to play a role of a detoxification product which protects the mammalian organism from arsenite toxicity.

Similar to arsenic, the underlying mechanism of the protective Se effect on Cd tissue distribution is attributed to the formation of a colloidal Cd–Se complex [116, 121]. The Cd–Se compound was first revealed in rat plasma in early in vitro and in vivo studies conducted by Gasiewicz and Smith [122]. The researchers reported that erythrocytes reduced selenite (SeO_3_^2−^) to selenide (H_2_Se), which was released to plasma forming a biologically inert, stable Cd–Se complex with Cd^2+^. Although it was suggested that the formation of the Cd–Se complex in plasma may reduce the Cd content in organs or tissues [116, 122], the fate and distribution pathways of the Cd–Se compound have not yet been elucidated. Recently, in a chemical experiment, sodium selenide (Na_2_Se) at toxic doses (50 μM) has been found to form insoluble complexes with various metal ions including Cd^2+^ [123]. In this report, the Cd–Se complex (containing equimolar amounts of Cd and Se) was stable in oxidizing conditions and nontoxic toward *Saccharomyces cerevisiae* cells [123]. As expected, the formation of the Cd–Se complex diminished the biological reactivity and toxicity of both elements.

## Increase in As Methylation Efficiency

Increasing the body’s As methylation capacity is another mode of action through which Se is assumed to decrease tissue accumulation of As and its toxic effects. For instance, inorganic As (As^III^ or As^V^) after ingestion is transformed to methylated species, i.e., methylarsonic acid (MMA^V^) and dimethylarsinic acid (DMA^V^), which are then excreted in the urine. Since the methylated pentavalent As species (MMA^V^ and DMA^V^) mentioned above are less toxic than inorganic forms, the methylation of As is generally regarded as a detoxification mechanism [124]. However, during this pathway, intermediate methylated forms, i.e., methylarsonous acid (MMA^III^) and dimethylarsinous acid (DMA^III^), are also produced and both of them are more toxic than arsenite [125]. Nevertheless, previous studies have shown that people who had lower capacity to fully methylate inorganic As to DMA^V^ had a higher risk of developing As-related diseases such as skin cancer [126], urothelial carcinoma [127], and peripheral vascular disease [128].

Many observational studies of human populations have evaluated the associations between the Se status and proportions of As metabolites, suggesting a protective effect of Se on increased As methylation efficiency. For example, a higher concentration of urinary Se was associated with an increased percentage of DMA^V^ and a decreased percentage of inorganic As in the urine of As-exposed Taiwanese adults [129] and pregnant women in Chile [124]. The findings in other adults with As exposure showed that plasma Se concentrations were inversely associated with total As levels in the blood and urine and the percentage of MMA^V^ in the blood and positively associated with the percentage of DMA^V^ in the blood. Simultaneously, plasma Se did not influence the As metabolite profile in the urine of the studied population [130]. Most recently, a case–control study demonstrated that higher plasma Se levels were associated with a lower percentage of MMA^V^ and a higher percentage of DMA^V^ in the urine of preschool children in Taiwan [131]. These results demonstrate that an optimal Se status may help to increase the body’s capability of As methylation. The mechanism through which Se is suggested to enhance As methylation involves the reductive thioredoxin (Trx) system. The Trx system is composed of thioredoxin reductase (TrxR, a selenoenzyme), Trx, and NADPH. It has been demonstrated that human arsenite methyltransferase (AS3MT, an enzyme which catalyzes methylation of As^III^ by using SAM as a methyl donor) uses Trx as a reductant for two intermediate reduction steps required during the As^III^ methylation pathway. Oxidized thioredoxin is regenerated by TrxR with electrons from NADPH [132]. Selenium deficiency decreases the activity of TrxR, which compromises the production of reduced Trx; consequently, this may impair As^III^ methylation catalyzed by AS3MT [130].

However, conflicting results have also been reported. For example, in children, Skröder Löveborn et al. [133] have found a positive correlation between the erythrocyte concentration of Se and the percentage of inorganic As and MMA^V^ and a negative relationship with the percentage of DMA^V^ in urine (suggesting Se-mediated inhibition of As methylation). Styblo and Thomas [134] pointed out that selenite at a 2-μM dose inhibited As^III^ methylation, increased cellular retention of inorganic As, and significantly increased the toxicity of As^III^, MMA^III^, and DMA^III^ toward primary rat hepatocytes. This can be explained by the fact that, especially in excessive doses in contrast to low doses, Se can suppress As methylation by competing for metabolic substrates such as GSH or SAM [62]. The precise mechanisms of the Se-mediated inhibition of As methylation have been described in the next-to-last chapter of this article.

## Antioxidant Effect

As already mentioned, Se-mediated antioxidative mechanisms are primary related with upregulation of Se-proteins, GPx and TrxR. Recent studies have reported decreased mRNA expression or activity of GPx and/or TrxR in animal tissues or cultured cells after exposure to arsenite [90–92, 100] and Cd [102, 104–106, 109]. As expected, co-exposure to arsenite and selenite or Cd and selenite significantly restored the activity of these enzymes and decreased arsenite- and Cd-mediated tissue and cellular oxidative damage, providing evidence that GPx and TrxR protect animal cells against arsenite and Cd toxicity.

In addition, most recent studies link the Se antioxidant effects against arsenite and Cd with the activation of the Nrf2 factor. Briefly, Nrf2 is a transcription factor accumulating in the nucleus in oxidative and electrophilic stress conditions, where it activates the transcription of ARE (antioxidant response element)-responsive antioxidant and detoxifying enzymes that promote cell survival [135]. While in mild and moderate oxidative stress conditions, the Nrf2 activity is stimulated as a beneficial adaptive response increasing the protective capacity of cells, high oxidative stress levels may inhibit the Nrf2 pathway and, thus, enhance the deleterious effects of toxic agents [135]. Accordingly, in a study conducted by Shafik and El Batsh [91], arsenite treatment downregulated the Nrf2 pathway and the activity of TrxR (ARE-responsive selenoprotein) in the liver of rats. Conversely, co-administration of selenite to arsenite-exposed rats partly reversed the decrease in Nrf2 expression and increased the TrxR activity, which had a protective effect against As-induced hepatotoxicity [91]. In addition, one in vitro study suggested that also the Nrf2 pathway may be involved in the regulation of apoptosis by Se and Cd mutual effects [105]. In this work, the investigators pointed out that Se triggered the activation of Nrf2, which in turn upregulated the transcription of ARE-responsive genes (NQO1, HO-1, GST, GCLC/M, which were downregulated by Cd) and finally reduced Cd-mediated apoptosis in cultured chicken hepatocytes [105]. This, however, contrasts with the results of an in vivo study, in which Se prevented Cd-induced apoptosis in chicken spleen, and the Nrf2 response pathway was rather downregulated in the presence of Se [106]. Notably, the two cited studies reflect the dual effects of Cd supplementation on Nrf2 regulation, namely inhibition of Nrf2-related genes as observed in vitro in hepatocytes [105] and their activation as reported in vivo for spleen [106]. The discrepancy between the two studies could be explained by, e.g., the differences between the in vitro and in vivo environments and the dissimilar target cell types (hepatocytes versus splenic cells). In addition, as already mentioned, the intensity of oxidative stress may either activate (moderate oxidative stress) or inhibit (high oxidative stress) Nrf2 activity [135]. Nevertheless, in both studies, Se counteracted the Cd effects on the Nrf2 activity. Interestingly, an important role of TrxR1 in Nrf2 regulation was proposed by Cebula et al. [136]. It was observed that inhibition of the catalytic activity of TrxR1 (for example by Se deficiency), which in turn may diminish the capacity in TrxR1-mediated processes, leads to Nrf2 activation. In contrast, via thioredoxin-mediated reduction of cysteine residues in Nrf2 or Keap1, active TrxR1 functions as a negative regulator of Nrf2 preventing its activation. It can be hypothesized that longer Se supplementation (90 days), such as that applied in a study conducted by Chen et al. [106], may be sufficient for fully activated TrxR1 to inhibit the Nrf2 pathway. Altogether, the effects of Se supplementation on Nrf2 regulation are complex and equivocal and possibly dependent on various conditions such as those associated with the redox state environment or the level of oxidative stress. Most recently, Se (as biogenic nanoselenium particles) alone activated the Nrf2 factor and increased the expression of its downstream genes such as thioredoxin reductase (Txnrd)-1, NADPH dehydrogenase (NQO)-1, heme oxygenase (HO)-1, and Trx in porcine jejunum epithelial cells [137]. In that study, through activation of Nrf2 and its downstream genes, nanoselenium improved the cell redox state and protected against oxidative stress and apoptosis [137].

Summing up, it can be deduced that at least some protective effects of Se supplementation against arsenite or Cd such as the increased activity of antioxidant enzymes observed in many studies may be mediated through upregulation of the Nrf2 pathway.

## Symptoms of Se Overdose in Studies Examining the Effects of Se on As^III^ and Cd Toxicity

In the studies included in Tables 2 and 3, Se was supplemented mostly at doses selected on the basis of previous experiments, and in most cases, it was nontoxic. However, we identified certain exceptions in terms of Se effects on oxidative stress-related markers that can indicate signs of Se toxicity probably caused by overdose of this element. For example, one study [93], which examined SeMet effects on As^III^ toxicity in mice, reported that SeMet (2 ppm)-fortified diet given alone (without As) significantly elevated hepatic lipid peroxidation, compared to animals fed adequate Se diet (0.2 ppm of SeMet). The increased peroxidation of lipids in response to the SeMet-fortified diet obviously indicates SeMet toxicity. This result is in sharp contrast with other studies included in this review, in which Se efficiently prevented lipid peroxidation. In another study of the Se effects on Cd toxicity in chickens, Se at a dose of 2 mg kg^−1^ diet (as selenite) given alone (without Cd) significantly induced the expression of heat shock protein (Hsp) genes (Hsp40, Hsp60, and Hsp70) in neutrophils, compared to controls [107]. Since Hsp proteins are reported to be induced in cells in stress conditions [138], this result may indicate Se-mediated cellular damage. Interestingly, in the same study, co-administration of 2 mg Se kg^−1^ diet to Cd-exposed chickens significantly ameliorated the Cd-mediated stress, as shown by the reduced mRNA expression levels for Hsp40 and Hsp70, in comparison with Cd treatment alone. This result confirms the well-known paradox of Se effects, when apparently toxic doses of Se co-administered with other toxic elements may help to counteract their toxic effects [52].

The mechanisms of Se-mediated toxicity have not been fully elucidated. It is known, however, that some Se compounds such as selenite, selenocysteine, or methylseleninic acid are redox-active molecules that generate ROS and induce oxidative stress [139]. For example, selenite has long been reported to react with GSH, producing the following compounds: selenodiglutathione, glutathioselenol, hydrogen selenide, and finally elemental Se (Se^0^) with concomitant generation of ROS [140]. As shown by Tarze et al. [141], hydrogen selenide was the most toxic product of selenite and GSH reaction against *Saccharomyces cerevisiae* and possibly directly accounted for the toxicity of selenite. The authors proposed that selenide promotes continuous consumption of intracellular reduced glutathione, thus leading to oxidative stress [141]. With regard to SeMet-induced toxicity, oxidative stress can also contribute to its deleterious effects in animal cells; however, the mechanism of its generation in the presence of this selenoamino acid has not yet been explored [142]. Moreover, a recent study has shown that SeMet toxicity in a *Saccharomyces cerevisiae* model is mediated by SeMet-induced accumulation of protein aggregates [143]. The report suggested that SeMet is transformed to SeCys followed by SeCys misincorporation into proteins instead of Cys resulting in protein aggregation. A similar mechanism may also be responsible for SeMet toxicity in other organisms [143].

Another important aspect is that Se, especially when overdosed, can enhance the deleterious effects of inorganic As by inhibition of As methylation, as described by Sun et al. [62]. Since selenite and arsenite share similar metabolic pathways, which require the presence of GSH and SAM (a methyl donor), a higher concentration of selenite can compete with arsenite for the availability of GSH and SAM and, consequently, inhibit the formation of methylated As metabolites. This in turn increases the cellular retention of more toxic inorganic and monomethylated As forms [134]. In addition, the inhibition of As methylation in the presence of Se is often linked with the function of AS3MT. Studies demonstrated that selenite inhibited the methylation of arsenite by recombinant rat AS3MT in a competitive manner, probably through direct interaction between selenite and the enzyme [144]. Further studies have shown that selenite inhibited the activity of recombinant human AS3MT, and the mechanism of inhibition probably involved the interaction of selenite with cysteines in the structural residues of the enzyme [145]. Overall, these findings demonstrate that excessive selenite can adversely affect the metabolism of inorganic As through decreasing the GSH and SAM levels and suppressing the activity of AS3MT.

## Conclusions and Future Directions

**Fig. 3:**
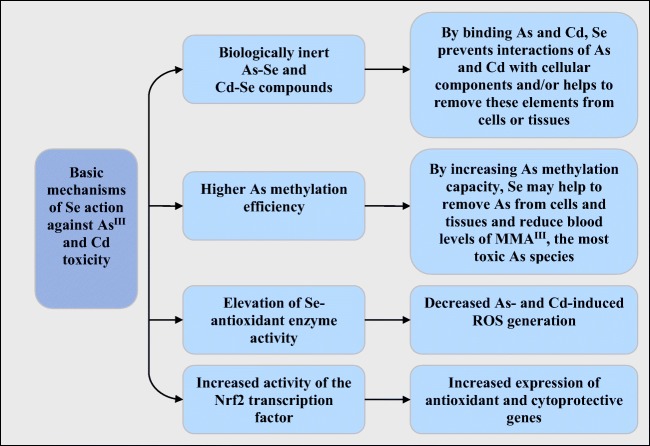
Scheme showing the direct mechanisms of Se action against As^III^ and Cd toxicity observed in animal and cell culture studies

Oxidative damage is one of the major mechanisms underlying As^III^ and Cd-induced cell injury. Therefore, one of the therapeutic strategies to counteract their toxicity in vivo is to use various antioxidants. Based on recent experimental studies, Se treatment during As^III^ or Cd intoxication can mitigate the deleterious effects of both toxic elements toward animal cells and tissues. As shown in Tables 2 and 3, co-administration of Se (mostly as Na_2_SeO_3_) with As^III^ and Cd prevented, e.g., lipid peroxidation, apoptosis induction, and histopathological changes and decreased As and Cd accumulation, compared to As^III^ or Cd treatment alone. The findings of recent papers support the notion that the major mechanisms of Se protection against As^III^ and Cd include (1) cleansing tissues and cells from As and Cd accumulation (possibly via excretion of As–Se and Cd–Se compounds and/or increasing As methylation capacity) and (2) upregulation of antioxidant selenoprotein synthesis (summarized in Fig. 3). Furthermore, as shown in the literature, stimulation of the Nrf2 pathway may be an additional mechanism through which Se can decrease As^III^ and Cd toxicity. Chronic or high-dose exposure to As^III^ or Cd may cause suppression of the Nrf2 pathway and decrease the capacity of cells to counteract the damaging effects induced by these elements. In contrast, transient activation of the Nrf2 pathway by dietary levels of Se upregulated the expression of a considerable group of antioxidant and detoxifying enzymes and proteins such as GST, HO-1, GCLC, SOD, or TrxR, which in the end antagonized the toxic As and Cd effects (Fig. 3).

However, some possible signs of Se toxicity in experimental animals can also be identified during management of As^III^ or Cd intoxication. This certainly reflects a dose of unpredictability of the Se effects, most probably caused by the very narrow line between therapeutic and toxic doses of Se compounds. The duality of Se effects (beneficial versus toxic) is well known and can be a hindrance in the therapeutic use of Se against As^III^ and Cd. Application of the nanoform of Se could be a promising strategy for reinforcement of the protective capacities of Se with minimization of the risks of Se-related deleterious activity. Nanoscale Se arouses scientists’ interest due to its higher bioavailability and lower toxicity, compared to ordinary Se forms [146]. For instance, selenite treatment in a mouse model was more toxic than nanoselenium in terms of deleterious effects on mice growth, liver functions, and hepatic lipid peroxidation [147]. Simultaneously, in this study, both nanoselenium and selenite were equally potent in increasing the GPx and GST activity in the liver of mice [147]. In another study, nanoselenium was found to be less toxic than SeMet in inducing liver toxicity in mice [148], although both nanosized Se and SeMet increased the liver and kidney GPx and TrxR activity with similar efficacy [148]. There are reports on the protective effects of nanoselenium against various toxic elements such as lead [149] and chromium (VI) [150] as well as the ameliorative action of nanoselenium against As^III^ already mentioned in earlier chapters [99]. Altogether, nanoselenium appears as a very attractive form of Se for the treatment of As^III^ or Cd intoxication, but more studies on this topic are needed.

Additionally, the use of Se in combination with plant-derived antioxidants can provide another interesting item for study. In the presence of some plant-derived agents, Se was shown to exert enhanced protective activity. For example, a combination of selenite with *Punica granatum* fruit extracts was more effective against As^III^-induced hepatotoxicity in rats than selenite alone [91]. The findings in this study indicated that the synergistic ameliorative activity of selenite and *P. granatum* exerted an effect on As^III^-mediated hepatic histopathological changes, lipid peroxidation, TrxR activity, and Nrf2 gene expression [91]. Similarly, the combination of selenite and polyphenol curcumin in utero provided more effective protection of mouse skin stem cells against As^III^-induced disruption, compared to individual treatment with selenite or curcumin [151]. The possible mechanisms of protection included de novo GSH biosynthesis and activation of the Nrf2 transcription factor [151].

In light of the recent concerns over potential negative impacts of excessive Se intake, the aforementioned alternatives, i.e., nanoscale Se and co-administration of Se with plant antioxidants, appear as promising strategies for reducing the therapeutic dose of Se in the treatment of As^III^ and Cd toxicity.
